# Truncated forms of RUNX3 Unlike Full Length Protein Alter Cell Proliferation in a TGF-β Context Dependent Manner

**Published:** 2017

**Authors:** Narges Rahmanian, Parastoo Tarighi, Mehdi Gharghabi, Maryam Torshabi, Ghorban Ali Tarfiei, Taiebeh Mohammadi Farsani, Seyed Naser Ostad, Mohammad Hossein Ghahremani

**Affiliations:** a *Department of Molecular Medicine, School of Advanced Technologies in Medicine, Tehran University of Medical Sciences, Tehran, Iran. *; b *Department of Medical Biotechnology, Faculty of Allied Medicine, Iran University of Medical Sciences, Tehran, Iran.*; c *Department of Toxicology and Pharmacology, Faculty of Pharmacy, Tehran University of Medical Sciences, Tehran, Iran.*; d *Department of Dental Biomaterial, Dental School, Shahid Beheshti University of Medical Sciences, Tehran, Iran.*; e *Department of Medical Biotechnology, School of Advanced Technologies in Medicine, Tehran University of Medical Sciences, Tehran, Iran.*

**Keywords:** Runt, Cancer, TGF-β, Domain Analysis, Apoptosis

## Abstract

The Runt related transcription factors (RUNX) are recognized as key players in suppressing or promoting tumor growth. RUNX3, a member of this family, is known as a tumor suppressor in many types of cancers, although such a paradigm was challenged by some researchers. The TGF-β pathway governs major upstream signals to activate RUNX3. RUNX3 protein consists of several regions and domains. The Runt domain is a conserved DNA binding domain and is considered as the main part of RUNX proteins. Herein, we compared the effects of Runt domains and full-Runx3 in cell viability by designing two constructs of Runx3, including N-terminal region and Runt domain. We investigated the effect of full-Runx3, N-t, and RD on growth inhibition in AGS, MCF-7, A549, and HEK293 cell lines which are different in TGF-β sensitivity, in the absence and presence of TGF-β. The full length RUNX3 did not notably inhibit growth of these cell lines while, the N-t and RD truncates showed different trends in these cell lines. Cell proliferation in the TGF-β impaired context cell lines (AGS and MCF-7) significantly decrease while in the A549 significantly increase. On the other hand, transfection of N-t and RD did not considerably affect the cell proliferation in the HEK293.Our results show that full-lenght RUNX3 did not affect the cell viability. Conversely, the N-t and RD constructs significantly changed cell proliferation. Therefore, therapeutic potentials for these truncated proteins are suggested in tumors with RUNX proteins dysfunction, even in the TGF-β impair context.

## Introduction

The Runt related proteins (RUNX) are transcription factors with crucial roles in orchestrating the cell proliferation and differentiation in all stages of cell growth and development. Moreover, these proteins are known as key players in suppressing or promoting tumor growth (1-4). These transcription factors are context dependent in regulation of target genes. In fact, RUNX acts as a scaffold, employing other cofactors to activate or repress the promoter of interest. Thus, the consequent effect of RUNX on the cell proliferation and differentiation depends on the presence or absence of other cofactors in the cell-context (5-7). In mammals, three RUNX genes have been identified, including RUNX1, RUNX2 and RUNX3. RUNX3 is known as an important tumor suppressor in many types of cancers (8-13).The tumor suppressor role of RUNX3 was first reported by Ito and colleagues in 2002 (8) and many other papers have further supported this claim although such a paradigm was challenged afterwards by Groner team and other groups (14-16). Despite this discrepancy, it has been appeared that exogenous expression of the RUNX3 in tumor cells induces apoptosis and cell cycle arrest, reduces tumor bulk and vascularity, inhibits metastasis, and increases sensitivity of tumor cells to the chemotherapy (8, 17-20). RUNX3 orchestrate tumor suppression in a various signaling pathway crosstalk.When RUNX3 is in its inactive form, the Runt domain, and activation domain are masked by inhibitory regions and domain, while some signaling pathways, including TGF-β, which is one of the most crucial pathways, activate RUNX3 through certain cofactors and change the conformation of it. So that the RD and AD are exposed to DNA and other cofactors (5, 21, 22). In this pathway SMADs and RUNX3 have close cooperation to induce the expression of genes which is involved in apoptosis or cell cycle arrest. Accordingly, In many experiments the co-expression of SMAD3 or SMAD4 (the important proteins in TGF-β signaling) or TGF-β with RUNX3 has been exploited to reinforce RUNX3 efficiency in inhibition of tumor growth (23). In many tumors TGF-β pathway is impaired by inactivation of some component in this cascade (24-26). Hence, in such tumors, RUNX3 could not be introduced as a potential therapeutic agent.

RUNX3 with 415 amino acids consists of several domains, each of which interacts with various proteins to regulate RUNX activity in a spatio-temporal manner. The Runt domain (RD) which is located in the N-terminal part of RUNX proteins is a conserved DNA binding domain (128aa) with more than 90% identity among three RUNX genes, which binds to a specific motif in DNA (27). At the C-terminal, the most important regions include the transactivation domain (AD) and inhibition domain (ID). Transactivation domain binds to different cofactors and makes various combinations of transcription factors for activation of specific promoter. Inhibition domain lies in the C-terminal of activation domain and inhibits RUNX3 activity by masking the activation domain or binding to some inhibitory proteins (5, 7, 23, 28-31).The Runt domain as a conserved DNA binding domain has been considered the main part of RUNX proteins since, only this part binds to a specific motif in DNA (32). Furthermore, Runt domain contributes to nuclear localization and is able to translocate to the nucleus and bind to DNA with stronger affinity compared to the full protein (29). 

**Figure 1:**
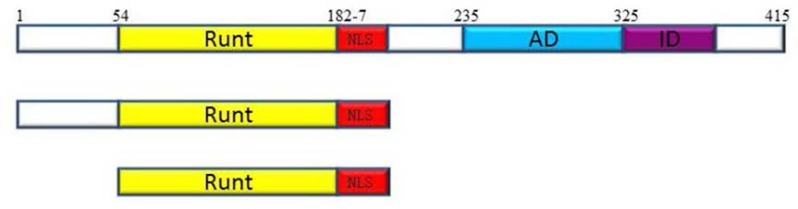
Schematic illustration of the structure of full-length RUNX3(4) and its deletion derivatives. The numbers represent the positions of amino acids. AD: transcription activation domain, ID: transcription inhibition domain, NLS: nuclear localization signal

Of further relevance, it is possible to assume that N-terminal region and Runt domain of the full protein, which do not contain inhibition domain or negative regulatory region are capable to inhibit cell growth in the absence of activating pathways. To address this hypothesis, we investigated the role of N-terminal region of the RUNX3 protein in tumor growth suppression by constructing two truncated forms of the Runx3: N-terminal region (1-187aa), and Runt domain (54-187) (Figure.1). Since TGF-β is known as an important factor for RUNX3 activation, we investigated the effect of full-Runx3, N-t, and RD on growth inhibition in AGS, MCF-7, A549, and HEK293 cell lines which are different in TGF- β sensitivity, in the absence or presence of TGF- β.

Our findings indicate that in the absence and presence of TGF-β exogenous RUNX3 could not provoke notable cell death in different cell lines, whereas the N-t (1-187) and RD (54-187) constructs, did significantly inhibit cell proliferation in AGS and MCF-7 cell lines with impaired TGF-β pathway.

## Experimental

*Cell culture and transfection*

The human gastric adenocarcinoma AGS cell-line (Pasteur Institute Cell Bank, Iran), human embryonic kidney HEK 293 cell-line(C10139,IBRC, Iran), and human breast adenocarcinoma MCF-7 cell-line (DSMZ, Braunschweig, Germany) were cultured and maintained in RPMI-1640 medium (Euroclone, EU).The human lung carcinoma A549 cell line (C10080, IBRC, Iran) was cultured and maintained in DMEM (Euroclone, EU) containing 10% FBS (Fetal Bovine Serum) (Gibco, UK) and 1% penicillin-streptomycin (Biosera, UK) in a humidified atmosphere at 37 °C and 5% CO_2_. Cells were seeded for 24h prior to transfection to reach 70-90% confluency. After 24 h. the cell lines were transiently transfected with PolyFect® transfection reagent (Qiagen, Germany) according to the manufacturer’s instructions.

Human recombinant TGF-β 1 was purchased from AbcamBiotech (Cambridge, UK). Twenty-four h. after transfection, the medium were exchanged in the absence or presence of 10ng/mL of TGF-β.

*Plasmid Constructs*

The type I isoform of full-length RUNX3 (NM_004350) and RUNX3 truncated mutants, N-t (1-187) and RD (54-187) were amplified by PCR with a C-terminus Histidin-tagged and cloned into the pcDNA3 vector. The final constructs were verified by restriction enzyme digestion and sequencing. The primer sequences have been shown in table 1.

*Cell Proliferation Assay*

To determine the effects of RUNX3 and its constructs on cell proliferation, MTT assay was carried out in three independent experiments. The cells were seeded in 96-well plate at 2×10^4 ^cells per well in quadruple. Following 24 h. incubation, cells were transfected using PolyFect®. Cell viability was measured using MTT 3-(4, 5-dimethylthiazol-2-yl) 2, 5-diphenyltetrazolium bromide (0.5mg/m), 24 and 48 h. after transfection. The formazan crystals were formed after incubation for 4 h. at 37 °C and then dissolved in DMSO. The optical density (OD) was measured using microplate reader (BioTek, USA) at 570 nm, with 690 nm as a reference wavelength. The change in viability was calculated as percent viability compared to that of the control group (empty vector). 

*Statistical analysis*

All data were presented as mean ± SEM (three independent experiments, n=3). The results were analyzed using one-way ANOVA following Tukey post-test. P-values< 0.05 were regarded as statistically significant.

## Results

*Assessment of Transfection efficiency*

The conditions of transfection were optimized by changing the number of cell plated, amount of plasmids and Polyfect reagent. The efficiency of transfection was assessed by transfection of pcDNA3-EGFP to the cells. The transfected cells, were evaluated 24 h. and 48 h. after transfection for the expression of EGFP by fluorescence microscopy. 

**Figure 2:**
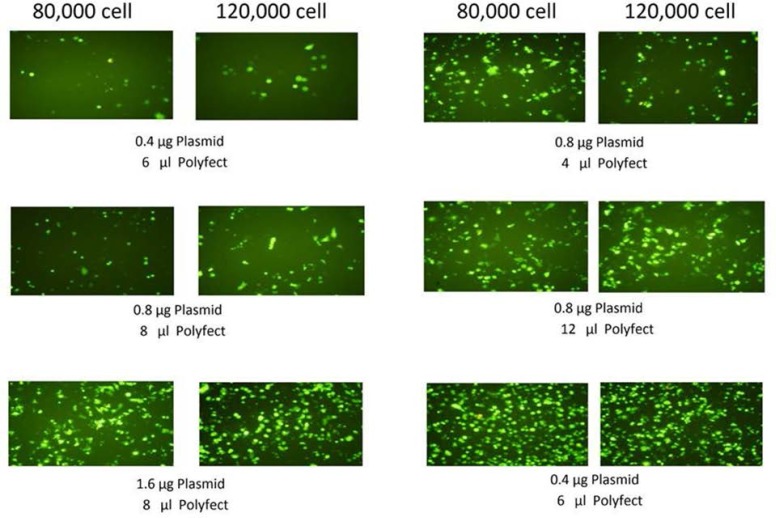
AGS cells were transfected by Polyfect reagent using different number of cellS and different amount of EGFP-plasmid and Polyfect reagent. 24 h. after transfection cells were examined for transfection efficiency by fluorescence microscopy

**Figure 3:**
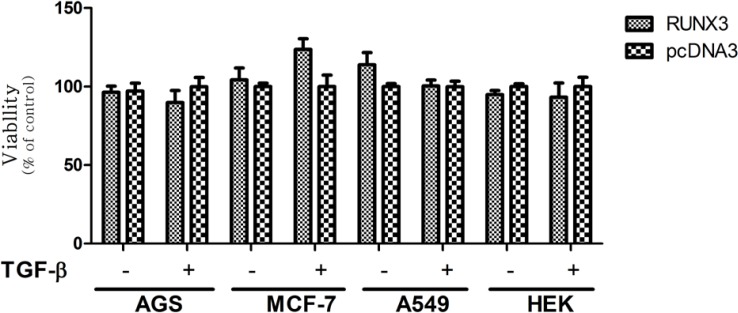
Effect of full-RUNX3 on Cell Viability in the Absence and Presence of TGF-β in AGS, MCF-7, A549, and HEK cell lines. Cells were transfected with RUNX3. 24 h. after transfection, the medium of the transfected cells was exchanged in the absence or presence of 10ng/mL TGF-β. After 48 h. cell proliferation was evaluated by MTT assay. Empty vector (pcDNA3) was used as a control. Data presented as Mean ± SE of three independent experiments

**Figure 4:**
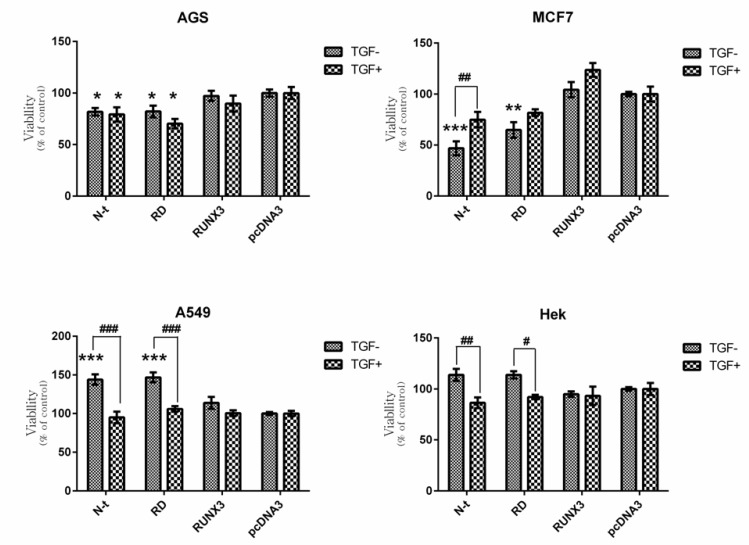
Effect of RUNX3, N-t, and RD on Cell Viability in the Absence and Presence of TGF-β. Cells were transfected with RUNX3, N-t, and RD. 24 h. after transfection, the medium of the transfected cells was exchanged in the absence or presence of 10ng/mL TGF-β. After 48 h. cell proliferation was evaluated by MTT assay. a) AGS b) MCF-7 c) A549 d) HEK293cell line. Empty vector (pcDNA3) was used as a control. Data presented as Mean ± SE of three independent experiments (n=3, * and # p<0.05, ** and ## p<0.01, ***and ### p<0.001). The sign of * correspond with construct vs pcDNA3 and the sign of # correspond with comparison of the same construct with and without TGF-β

**Table 1:** Primers for PCR amplifications of RUNX3 constructs

**PCR Product Name**	**Forward/Reverse**	**Primer Sequence**
RUNX3, N-t (1-187)	Forward	5´-AAGGAAAGAATTCGAACCATGCGTATCCCCGTAGAC-3´
RD (54-187)	Forward	5´-AGTGGGTACCATGCACGCAGGCGA-3´
RUNX3	Reverse	5´-AATCTAGATCTCAATGATGATGATGATGATGGTGAGGCCG-3´
N-t (1-187), RD (54-187)	Reverse	5´-ATTTGCGGCCGCATTAGTGGTGGTGGTGATGGTGCAGCTTCTG-3´

Figure 2 shows the effect of different condition of transfection (amount of cell, plasmid and Polyfect) on EGFP expression in AGS cell line. Based on EGFP expression results, the optimized condition including 120000 cells, 1.6 µg DNA and 8 µL Plyfect in a well of 24-well cell culture plate were used for subsequent transfections.

*Cell Proliferation Analysis of AGS, HEK, A549, and MCF-7 in the Absence and Presence of TGF-β*

The effects of full-RUNX3, N-t and RD constructs on cell proliferation in the absence or presence of TGF-β was carried out in AGS, MCF-7, A549, and HEK 293 cell lines. These cell lines are different in the functionality of TGF-β pathway. AGS cell line is resistant to TGF-β, whereas MCF-7, A549, and HEK 293 cell lines are sensitive to TGF-β (33-35). 

In our results, the RUNX3 did not notably induce growth inhibitory effects in all applied cell lines in the presence or absence of TGF-β (Figure 3). These findings are in contrast to previous studies indicating the growth inhibitory effect of RUNX3 in these cell lines (36-38). On the other hand, truncated forms of RUNX3 displayed different patterns of proliferation in each cell-line. In AGS cell line the N-t (1-187) and RD (54-187) constructs significantly inhibited cell proliferation, however, the presence or absence of TGF-β did not cause any significant difference in this cell-line (Figure 4a). In MCF-7 cell-line, in the absence and presence of TGF-β, the N-t and RD constructs decreased cell proliferation. These effects were higher in the absence of TGF-β (Figure 4b). In A549, in the absence of TGF-β, the N-t, and RD constructs increased cell proliferation about 40% and in the presence of TGF-β, none of the constructs could show any difference in comparison with the control (Figure 4c). In HEK cells, in the absence of TGF-β, the N-t, and RD constructs slightly increased cell proliferation, while in the presence of TGF-β, the growth pattern was changed, leading to a mild decrease in cell proliferation (about 10%) in (Figure 4d). 

## Discussion

Previous studies have pointed to the growth inhibitory effect of RUNX3 in different cell lines, including AGS, MCF-7, A549, and HEK 293(36-38). However, with regard to our results, the RUNX3 did not notably induce growth inhibition in these four cell lines in the presence or absence of TGF-β (Figure 3). Furthermore, the N-t and RD truncates showed different trends in these cell lines. Cell proliferation in the AGS and MCF-7 significantly decrease while in the A549 significantly increase. On the other hand, transfection of N-t and RD did not considerably affect the cell proliferation in the HEK293.

Given the above mentioned results stated thus far, a number of questions are rising:

Why RUNX3, which is known as an important tumor suppressor, does not exert any notable growth inhibitory effect in our cell lines?

Why N-t and RD constructs have a significant effect on cell proliferation, while the full-RUNX3 does not exert a such effect?

Why N-t and RD constructs provoke divergent effects on each cell-line?

Why each cell-line shows different behaviour in the presence of TGF-β?

For the first time, Ito team established that Runx3 was expressed in the glandular stomach epithelial cells, and Runx3 null gastric mucosa developed hyperplasia owing to the promotion of proliferation and suppression of apoptosis in epithelial cells. Consequently, they proposed that RUNX3 is a tumor-suppressor gene causally involved in gastric carcinogenesis (8). After publishing these data, many other papers substantiated the onco-suppressory role for RUNX3 in gastric and other tissues. By contrast, Groner team, which are pioneer in RUNX researches ruled out Itoʼresults. They generated RUNX3 ^-^/^-^ mice, which did not show any early-onset gastric hyperplasia and did not develop gastric carcinoma (14, 39, 40). They also assessed RUNX3 expression by various techniques, including IHC with eight different anti-RUNX3 antibodies and showed that RUNX3 was not even transiently expressed at any time point in the gastrointestinal (GI) tract epithelium (14). Furthermore, in their previous study of RUNX expression pattern during embryogenesis, they demonstrated that Runx3 expression was conﬁned to mesenchymal elements, whereas Runx1 was expressed in both epithelium and mesenchyme cells (41). They concluded that absence of RUNX3 expression in normal epithelium undermines the tumor suppressor role for RUNX3. This paradigm was supported by Cravalho *et al*., who likewise reported lack of RUNX3 expression in human normal GI tract epithelium (16). Moreover, Friedrich *et al.* examined RUNX3 expression in gastric biopsies from 105 patients. The IHC results indicated RUNX3 protein expression only in infiltrating leukocytes, but not in the gastric epithelium (15). Additionally, Itoʼs lab could not repeat their results on RUNX3-lacZ staining in GI tract (42). All the cell lines we used in this study are epithelial cells. Considering Gronerʼ results, RUNX3 is not expressed frequently in epithelial cells and hence, we cannot anticipate a tumor suppressor role for RUNX3 in these cell lines. On the other hand, RUNX is not merely a strong transcription factor, rather it can recruit other cofactors to exert its function as a scaffold (5). This notion can partly justify why we did not observe any significant effect of RUNX3 on cell proliferation. If so, one can ask why the N-t and RD constructs of RUNX3 have significant effects on cell proliferation, while the full-RUNX3 does not induce such effect.

The Runt domain is a conserved DNA binding domain (128aa) with more than 90% identity among three RUNX genes. This domain is considered as the main part of RUNX proteins since, only this part binds to a specific motif in DNA (32). Furthermore, Runt domain also contributes to nuclear localization and is able to translocate to the nucleus and bind to DNA with stronger affinity compared to the full protein (29). Although the activation domain is responsible for modulating RUNX function through interaction with certain cofactors, the exposed Runt domain by itself can interact with many cofactors, including Ets, C/EBP, and cbfb which consequently can regulate a number of RUNX functions regarding the cell context (5, 43, 44). Accordingly, the N-terminal constructs of the RUNX can interfere with the activity of all three mammalian RUNX proteins due to its high identity among three RUNX genes as well as its higher affinity for the consensus DNA binding site. Considering these data, the N-t and RD might bind to all three RUNX proteins’ DNA binding site and interfere with or mimic the activity of RUNX1 and RUNX2 in addition to the RUNX3. 

RUNX transcription factor participates in some biological activities of TGF-β signalling (45). TGF-β signalling pathway has a major role in the regulation of cell proliferation and differentiation which includes both tumor-suppressor and proto-oncogene arms. Even in the same cells, the response of TGF-β pathway differs depending on the environmental factors and cellular context. Hence, TGF-β may act as tumor suppressing or tumor promoting factor in cancer development. It has been generally established that TGF-β is a tumor-suppressor in the early stages of carcinogenesis, whereas is a tumor-promoter at late stages in tumor progression (46-48). RUNX proteins take part in both arms of the TGF–β pathway (45, 49). In fact, RUNX proteins show context-dependent manner in cell proliferation and differentiation; thus, are either proto-oncogenes or tumor-suppressors regarding their context (50).

Given the notion that the AGS cell-line is resistant to TGF-β, as anticipated, the presence or absence of TGF-β did not significantly alter cell proliferation in AGS transfected cells. Moreover, in this cell-line, the N-t and RD could significantly decrease cell proliferation (Figure 4a). It is known that RUNX1 is expressed in gastric epithelial cells and functions as a tumor-suppressor in gastric cancer cells (51). It is possible that these two truncated forms mimic the RUNX1 function in this context. Since AGS is resistant to TGF-β, this effect is likely due to other pathways that RUNX takes part in, such as FOXO and Wnt signalling (52, 53). Similar to gastric epithelium, RUNX1 is expressed in breast epithelial cells and acts as a tumor suppressor in breast cancer(3, 54). In MCF-7 cell-line, the N-t and RD constructs considerably decreased cell proliferation in the absence of TGF-β, while the interaction of TGF-β with these constructs reduced their inhibitory effect (Figure.4b). This means that in the presence of TGF-β, these truncated forms simultaneously show negative and positive levels of regulation in cell proliferation through different signalling pathways. In ERα-positive breast cancer cells like MCF-7, the tumor suppressor arm of TGF-β is impaired while, the proto-oncogene arm is functional; thus TGF-β promotes tumor development (34). RUNX1 in TGF-β signalling pathway can participate in both tumor-suppressor and tumor-promoter arms. Additionally, RUNX1 can exert the tumor suppressor role in breast cancer through other pathways, such as FOXO (34, 55). Consequently, the N-t and RD truncates, like RUNX1 can perhaps take part in pathways, such as FOXO in the absence of TGF-β, while in the presence of TGF-β, beside FOXO pathway, can play a weaker role in tumor-promoter arm of TGF-β.

Interestingly, the N-t and RD constructs could notably provoke cell proliferation in A549 cells (Figure 4c). Consistent with this finding, it has been reported that RUNX2 plays pivotal role in tumorogenicity in non-small cell lung cancer (NSCLC) (56, 57). Hence it is tempting to speculate that the N-t and RD perhaps mimic the RUNX2 function in NSCLC A549 cell line. On the other hand, the effect of these two constructs was completely blocked by TGF-β. In line of our result, Kang *et al*.(58) have demonstrated that TGF-β can block RUNX2 function, which can justify our results.HEK is a non-tumorogenic embryonic cell-line possessing the intact signalling network for RUNX and TGF-β pathways. Consequently, transfection of N-t and RD did not considerably affect the cell proliferation either in the presence or absence of TGF-β (Figure 4d).

## Conclusion

Whiles RUNX3 cannot notably suppress cancer cell proliferation; the N-t and RD constructs significantly suppress cell growth even in the TGF-β impaired context of AGS and MCF-7 cell lines. The N-terminal constructs of RUNX3 (N-t and RD) contain the conserved Runt domain with more than 90% identity among RUNX genes. Consequently, they might mimic the activity of all three mammalian RUNX proteins. Since many tumors are resistant to TGF-β, therapeutic potentials for these truncated proteins are suggested in tumors with TGF-β impairment and RUNX proteins dysfunction. These truncated RUNX3 proteins could be utilized in gene therapy or designing small molecule inhibitors (59). However the cellular context and environmental factors can influence on the regulation of RUNX target genes. Therefore, to further explore the use of these truncated forms of RUNX proteins in cancer therapy, one should propel the research in the context of tumors and its cellular signalling conditions using high-throughput techniques and system biology.

## References

[1] Speck NA, Stacy T, Wang Q, North T, Gu T-L, Miller J, Binder M, Marín-Padilla M (1999). Core-binding factor: a central player in hematopoiesis and leukemia. Cancer res..

[2] Levanon D, Groner Y (2004). Structure and regulated expression of mammalian RUNX genes. Oncogene.

[3] Blyth K, Cameron ER, Neil JC (2005). The RUNX genes: gain or loss of function in cancer. Nature. Rev. Cancer.

[4] Chuang LS, Ito K, Ito Y (2013). RUNX family: Regulation and diversification of roles through interacting proteins. Int. J. Cancer.

[5] Kagoshima H, Shigesada K, Satake M, Ito Y, Miyoshi H, Ohki M, Pepling M, Gergen P (1993). The Runt domain identifies a new family of heteromeric transcriptional regulators. Trends. Genet..

[6] Bangsow C, Rubins N, Glusman G, Bernstein Y, Negreanu V, Goldenberg D, Lotem J, Ben-Asher E, Lancet D, Levanon D, Groner Y (2001). The RUNX3 gene-sequence, structure and regulated expression. Gene.

[7] Pande S, Ali SA, Dowdy C, Zaidi SK, Ito K, Ito Y, Montecino MA, Lian JB, Stein JL, van Wijnen AJ, Stein GS (2009). Subnuclear targeting of the Runx3 tumor suppressor and its epigenetic association with mitotic chromosomes. J. Cell. Physiol..

[8] Li QL, Ito K, Sakakura C, Fukamachi H, Inoue K, Chi XZ, Lee KY, Nomura S, Lee CW, Han SB, Kim HM, Kim WJ, Yamamoto H, Yamashita N, Yano T, Ikeda T, Itohara S, Inazawa J, Abe T, Hagiwara A, Yamagishi H, Ooe A, Kaneda A, Sugimura T, Ushijima T, Bae SC, Ito Y (2002). Causal relationship between the loss of RUNX3 expression and gastric. Cancer.Cell.

[9] Yanagawa N, Tamura G, Oizumi H, Takahashi N, Shimazaki Y, Motoyama T (2003). Promoter hypermethylation of tumor suppressor and tumor-related genes in non-small cell lung cancers. Cancer Sci..

[10] Xiao WH, Liu WW (2004). Hemizygous deletion and hypermethylation of RUNX3 gene in hepatocellular carcinoma. World J. Gastroenterol..

[11] Ku JL, Kang SB, Shin YK, Kang HC, Hong SH, Kim IJ, Shin JH, Han IO, Park JG (2004). Promoter hypermethylation downregulates RUNX3 gene expression in colorectal cancer cell lines. Oncogene.

[12] Wada M, Yazumi S, Takaishi S, Hasegawa K, Sawada M, Tanaka H, Ida H, Sakakura C, Ito K, Ito Y, Chiba T (2004). Frequent loss of RUNX3 gene expression in human bile duct and pancreatic cancer cell lines. Oncogene.

[13] Kim EJ, Kim YJ, Jeong P, Ha YS, Bae SC, Kim WJ (2008). Methylation of the RUNX3 promoter as a potential prognostic marker for bladder tumor. J. Urol..

[14] Levanon D, Bernstein Y, Negreanu V, Bone KR, Pozner A, Eilam R, Lotem J, Brenner O, Groner Y (2011). Absence of Runx3 expression in normal gastrointestinal epithelium calls into question its tumour suppressor function. EMBO. mol. med..

[15] Friedrich M, Rad R, Langer R, Voland P, Hoefler H, Schmid R, Prinz C, Gerhard M (2006). Lack of RUNX3 regulation in human gastric cancer. The j. pathol..

[16] Carvalho R, Milne AN, Polak M, Corver WE, Offerhaus GJA, Weterman MA (2005). Exclusion of RUNX3 as a tumour-suppressor gene in early-onset gastric carcinomas. Oncogene.

[17] Guo C, Ding J, Yao L, Sun L, Lin T, Song Y, Fan D (2005). Tumor suppressor gene Runx3 sensitizes gastric cancer cells to chemotherapeutic drugs by downregulating Bcl-2, MDR-1 and MRP-1. Int. J. Cancer.

[18] Hasegawa K, Yazumi S, Wada M, Sakurai T, Kida M, Yamauchi J, Hisatsune H, Tada S, Ida H, Nakase Y, Sakakura C, Hagiwara A, Chiba T (2007). Restoration of RUNX3 enhances transforming growth factor-beta-dependent p21 expression in a biliary tract cancer cell line. Cancer Sci..

[19] Nishina S, Shiraha H, Nakanishi Y, Tanaka S, Matsubara M, Takaoka N, Uemura M, Horiguchi S, Kataoka J, Iwamuro M, Yagi T, Yamamoto K (2011). Restored expression of the tumor suppressor gene RUNX3 reduces cancer stem cells in hepatocellular carcinoma by suppressing Jagged1-Notch signaling. Oncol. Rep..

[20] Torshabi M, Faramarzi MA, Tabatabaei Yazdi M, Ostad SN, Gharemani MH (2011). Runx3 Expression Inhibits Proliferation and Distinctly Alters mRNA Expression of Bax in AGS and A549 Cancer Cells. Iran. J. Pharm. Res..

[21] Ito Y (1999). Molecular basis of tissue-specific gene expression mediated by the runt domain transcription factor PEBP2/CBF. Genes Cells.

[22] Kim WY, Sieweke M, Ogawa E, Wee HJ, Englmeier U, Graf T, Ito Y (1999). Mutual activation of Ets-1 and AML1 DNA binding by direct interaction of their autoinhibitory domains. EMBO. J..

[23] Hanai J, Chen LF, Kanno T, Ohtani-Fujita N, Kim WY, Guo WH, Imamura T, Ishidou Y, Fukuchi M, Shi MJ, Stavnezer J, Kawabata M, Miyazono K, Ito Y (1999). Interaction and functional cooperation of PEBP2/CBF with Smads Synergistic induction of the immunoglobulin germline Calpha promoter. J. Biol. Chem.

[24] Chuang LS, Ito Y (2010). RUNX3 is multifunctional in carcinogenesis of multiple solid tumors. Oncogene.

[25] Yano T, Ito K, Fukamachi H, Chi XZ, Wee HJ, Inoue K, Ida H, Bouillet P, Strasser A, Bae SC, Ito Y (2006). The RUNX3 tumor suppressor upregulates Bim in gastric epithelial cells undergoing transforming growth factor beta-induced apoptosis. Mol.Cell. Biol..

[26] Ito K, Liu Q, Salto-Tellez M, Yano T, Tada K, Ida H, Huang C, Shah N, Inoue M, Rajnakova A, Hiong KC, Peh BK, Han HC, Ito T, Teh M, Yeoh KG, Ito Y (2005). RUNX3, a novel tumor suppressor, isfrequently inactivated in gastric cancer by protein mislocalization. Cancer Res..

[27] Fukushima-Nakase Y, Naoe Y, Taniuchi I, Hosoi H, Sugimoto T, Okuda T (2005). Shared and distinct roles mediated through C-terminal subdomains of acute myeloid leukemia/Runt-related transcription factor molecules in murine development. Blood.

[28] Zeng C, van Wijnen AJ, Stein JL, Meyers S, Sun W, Shopland L, Lawrence JB, Penman S, Lian JB, Stein GS, Hiebert SW (1997). Identification of a nuclear matrix targeting signal in the leukemia and bone-related AML/CBF-alpha transcription factors. Proc. Natl. Acad. Sci. U S A..

[29] Liu H, Carlsson L, Grundstrom T (2006). Identification of an N-terminal transactivation domain of Runx1 that separates molecular function from global differentiation function. J. Biol.Chem..

[30] Kanno T, Kanno Y, Chen LF, Ogawa E, Kim WY, Ito Y (1998). Intrinsic transcriptional activation-inhibition domains of the polyomavirus enhancer binding protein 2/core binding factor alpha subunit revealed in the presence of the beta subunit. Mol. Cell. Biol..

[31] Zhang YW, Bae SC, Huang G, Fu YX, Lu J, Ahn MY, Kanno Y, Kanno T, Ito Y (1997). A novel transcript encoding an N-terminally truncated AML1/PEBP2 alphaB protein interferes with transactivation and blocks granulocytic differentiation of 32Dcl3 myeloid cells. Mol. Cell. Biol..

[32] Crute BE, Lewis AF, Wu Z, Bushweller JH, Speck NA (1996). Biochemical and biophysical properties of the core-binding factor α2 (AML1) DNA-binding domain. j.Biol. Chem..

[33] Xu C-C, Wu L-M, Sun W, Zhang N, Chen W-S, Fu X-N (2011). Effects of TGF-β signaling blockade on human A549 lung adenocarcinoma cell lines. Molecular medicine reports..

[34] Ren Y, Wu L, Frost AR, Grizzle W, Cao X, Wan M (2009). Dual effects of TGF-β on ERα-mediated estrogenic transcriptional activity in breast cancer. Mol. cancer..

[35] Kim Y-W, Park J, Lee H-J, Lee S-Y, Kim SJ (2012). TGF-β sensitivity is determined by N-linked glycosylation of the type II TGF-β receptor. Biochem. J..

[36] Yamada C, Ozaki T, Ando K, Suenaga Y, Inoue K-i, Ito Y, Okoshi R, Kageyama H, Kimura H, Miyazaki M (2010). RUNX3 modulates DNA damage-mediated phosphorylation of tumor suppressor p53 at Ser-15 and acts as a co-activator for p53. J. Biol. Chem..

[37] Kang H-F, Dai Z-J, Bai H-P, Lu W-F, Ma X-B, Bao X, Lin S, Wang X-J (2013). RUNX3 gene promoter demethylation by 5-Aza-CdR induces apoptosis in breast cancer MCF-7 cell line. OncoTargets and therapy..

[38] Chi X-Z, Kim J, Lee Y-H, Lee J-W, Lee K-S, Wee H, Kim W-J, Park W-Y, Oh B-C, Stein GS (2009). Runt-related transcription factor RUNX3 is a target of MDM2-mediated ubiquitination. Cancer res..

[39] Levanon D, Groner Y (2009). Runx3-deficient mouse strains circa 2008: resemblance and dissimilarity. Blood Cells Mol. Dis..

[40] Lotem J, Levanon D, Negreanu V, Groner Y (2013). The False Paradigm of RUNX3 Function as Tumor Suppressor in Gastric Cancer.

[41] Levanon D, Brenner O, Negreanu V, Bettoun D, Woolf E, Eilam R, Lotem J, Gat U, Otto F, Speck N (2001). Spatial and temporal expression pattern of Runx3 (Aml2) and Runx1 (Aml1) indicates non-Fredundant functions during mouse embryogenesis. Mech. dev..

[42] Normile D (2011). Dispute over tumor suppressor gene Runx3 boils over. Science.

[43] Zhang D-E, Hetherington CJ, Meyers S, Rhoades KL, Larson CJ, Chen H-M, Hiebert SW, Tenen DG (1996). CCAAT enhancer-binding protein (C/EBP) and AML1 (CBF alpha2) synergistically activate the macrophage colony-stimulating factor receptor promoter. Mol. Cell. Bio..

[44] Ito Y (1996). Structural alterations in the transcription factor PEBP2/CBF linked to four different types of leukemia. J. cancer res. clin. onco..

[45] Miyazono K, Maeda S, Imamura T (2004). Coordinate regulation of cell growth and differentiation by TGF-β superfamily and Runx proteins. Oncogene.

[46] Derynck R, Akhurst RJ, Balmain A (2001). TGF-β signaling in tumor suppression and cancer progression. Nature genet..

[47] Ikushima H, Miyazono K (2010). Cellular context-dependent “colors” of transforming growth factor-β signaling. Cancer science.

[48] Akhurst RJ, Derynck R (2001). TGF-β signaling in cancer–a double-edged sword. Trends cell biol..

[49] Gonzàlez Juncà A (2013). Study of molecular mechanisms implicated in the TGF-beta oncogenic effect in Glioma.

[50] Coffman JA (2003). Runx transcription factors and the developmental balance between cell proliferation and differentiation. Cell. biol .intl..

[51] Fijneman RJ, Anderson RA, Richards E, Liu J, Tijssen M, Meijer GA, Anderson J, Rod A, O’Sullivan MG, Scott PM (2012). Runx1 is a tumor suppressor gene in the mouse gastrointestinal tract. Cancer science..

[52] Ito K (2011). RUNX3 in oncogenic and anti-oncogenic signaling in gastrointestinal cancers. J. cell. biochem..

[53] Torquati A, O’Rear L, Longobardi L, Spagnoli A, Richards WO, Beauchamp RD (2004). RUNX3 inhibits cell proliferation and induces apoptosis by reinstating transforming growth factor beta responsiveness in esophageal adenocarcinoma cells. Surgery.

[54] Chimge N, Frenkel B (2013). The RUNX family in breast cancer: relationships with estrogen signaling. Oncogene.

[55] Scheitz CJF, Tumbar T (2013). New insights into the role of Runx1 in epithelial stem cell biology and pathology. J. cell. biochem..

[56] Li H, Zhou R-J, Zhang G-Q, Xu J-P (2013). Clinical significance of RUNX2 expression in patients with nonsmall cell lung cancer: a 5-year follow-up study. Tumor. Biology.

[57] Hsu Y-L, Huang M-S, Yang C-J, Hung J-Y, Wu L-Y, Kuo P-L (2011). Lung tumor-associated osteoblast-derived bone morphogenetic protein-2 increased epithelial-to-mesenchymal transition of cancer by Runx2/Snail signaling pathway. j. Biol. Chem..

[58] Kang JS, Alliston T, Delston R, Derynck R (2005). Repression of Runx2 function by TGF-β through recruitment of class II histone deacetylases by Smad3. EMBO. j..

[59] Hajimahdi Z (2016). Small Molecules as Protein-Protein Interaction Inhibitors. Iran. J. Pharm. Res..

